# A tool for predicting overall survival in patients with Ewing sarcoma: a multicenter retrospective study

**DOI:** 10.1186/s12885-022-09796-7

**Published:** 2022-08-23

**Authors:** Wenle Li, Shengtao Dong, Yuewei Lin, Huitao Wu, Mengfei Chen, Chuan Qin, Kelin Li, JunYan Zhang, Zhi-Ri Tang, Haosheng Wang, Kang Huo, Xiangtao Xie, Zhaohui Hu, Sirui Kuang, Chengliang Yin

**Affiliations:** 1 Department of Orthopedic Surgery II, The Second Affiliated Hospital of Xi’an Jiao Tong University, Xi’an, 710004 China; 2 College of Life Science and Technology, Xi’an Jiaotong University, Xi’an, 710049 China; 3 Molecular Imaging and Translational Medicine Research Center, State Key Laboratory of Molecular Vaccinology and Molecular Diagnostics, Xiamen University, Xiamen, 361005 China; 4 Clinical Medical Research Center, Xianyang Central Hospital, Xianyang, 712099 China; 5 Department of Spine Surgery, Second Affiliated Hospital of Dalian Medical University, Dalian, 116000 China; 6 The Second Clinical College, Guangzhou University of Chinese Medicine, Guangzhou, 510000 China; 7 Intelligent Healthcare Team, Baidu Inc, Beijing, 100089 China; 8 Emergency Department, People’s Hospital of Ningxia Hui Autonomous Region, Yinchuan, 750000 China; 9 Department of Spine Surgery, Liuzhou People’s Hospital, Liuzhou, 545000 China; 10 Medical Big Data Research Center, PLA General Hospital, Beijing, 100853 China; 11 National Engineering Laboratory for Medical Big Data Application Technology, Chinese PLA General Hospital, Beijing, 100853 China; 12 School of Physics and Technology, Wuhan University, Wuhan, 430072 China; 13 Orthopaedic Medical Center, The Second Hospital of Jilin University, Changchun, 130000 China; 14 Neurology department, Xi’an jiaotong university 1st affiliated hospital, Xian, 71000 China; 15 Faculty of Medicine, Macau University of Science and Technology, Macau, 999078 China

**Keywords:** Ewing sarcoma, SEER database, Multicenter, Nomogram, Web calculator

## Abstract

**Objective:**

The aim of this study was to establish and validate a clinical prediction model for assessing the risk of metastasis and patient survival in Ewing's sarcoma (ES).

**Methods:**

Patients diagnosed with ES from the Surveillance, Epidemiology and End Results (SEER) database for the period 2010-2016 were extracted, and the data after exclusion of vacant terms was used as the training set (*n*=767). Prediction models predicting patients' overall survival (OS) at 1 and 3 years were created by cox regression analysis and visualized using Nomogram and web calculator. Multicenter data from four medical institutions were used as the validation set (*n*=51), and the model consistency was verified using calibration plots, and receiver operating characteristic (ROC) verified the predictive ability of the model. Finally, a clinical decision curve was used to demonstrate the clinical utility of the model.

**Results:**

The results of multivariate cox regression showed that age, , bone metastasis, tumor size, and chemotherapy were independent prognostic factors of ES patients. Internal and external validation results: calibration plots showed that the model had a good agreement for patient survival at 1 and 3 years; ROC showed that it possessed a good predictive ability and clinical decision curve proved that it possessed good clinical utility.

**Conclusions:**

The tool built in this paper to predict 1- and 3-year survival in ES patients (https://drwenleli0910.shinyapps.io/EwingApp/) has a good identification and predictive power.

**Supplementary Information:**

The online version contains supplementary material available at 10.1186/s12885-022-09796-7.

## Introduction

Ewing's sarcoma (ES) was first reported by the renowned pathologist James Ewing in 1922 [1], and the most frequent highly malignant bone tumor as a primary bone tumor second only to osteosarcoma with a peak incidence in the second decade of life. ES shows a significant age preference, with children and adolescents occupying the major age distribution of patients [2]. Previous studies have shown that tumors most often originate in the tubular bones of the extremities (46%), predominantly in the lower extremities, followed by the pelvis (25%), trunk including ribs and spinal trunk (22%), and other sites (6%), respectively [3]. The 5-year overall survival (OS) rate for patients with localized ES has increased from approximately 10% to 55%-65%, taking advantage of the application of a combination of surgery, chemotherapy and radiotherapy [3].

Limited by the low incidence of ES [2], researchers have difficulty recruiting a sufficient number of ES cases for their cohort studies. This obstacle is strengthened by the SEER database, which consists of 18 cancer registries covering approximately 30% of the total US population [4]. Meanwhile, the Nomogram is extensively used to assess tumor prognosis [5]. This reliable visual graph ensures that clinicians can conveniently and rapidly in get predictive results. By combining the incorporated predictors, the graph provides a personalized estimate of the risk of events, including the probability of disease recurrence or mortality, and is helpful in making scientifically sound clinical decisions and improving the prognosis of patients [6, 7]. In recent years, web calculators have become an emerging tendency in clinical predictive modeling, with the advantage of reducing the learning costs required to assess patient survival. Relying on a smartphone, users can efficiently and accurately calculate the risk for various patients [8].

Although investigators have never delayed exploring the prognosis of ES, this article was the first study to incorporate site-specific metastases to establish and externally validate data using a multicenter approach [9, 10]. This study extracted clinicopathologic, and treatment-informed ES patient data from SEER data, subsequently used statistically validated variables to build and validate a clinical prediction model that could be used to predict OS at 1 and 3 years of ES patients. The model was also externally validated using data from up to 4 medical institutions.

## Method

### Case screening

Since patient consent was not necessary to obtain access to the SEER database, this study used SEER * STAT (8.3.5) software to extract ES cases as the training group. Patients meeting the following criteria were included: (1) diagnosis of ES with ICD-O-3/WHO 2008 morphology code 9260; (2) complete clinical information including demographic characteristics (age, sex, race at diagnosis), tumor characteristics (primary site, tumor volume, TNM stage, distant metastases) and history of tumor treatment (surgery, radiotherapy, chemotherapy). Considering that the specific surgery, radiation and chemotherapy regimens were not included with the SEER database, all three treatments were classified as No or Unknown, and Yes.

The external validation group was obtained from four medical institutions including the Second Affiliated Hospital of Jilin University, the Second Affiliated Hospital of Dalian Medical University, Liuzhou People's Hospital, and Xianyang Central Hospital, respectively. Two senior pathologists at each center were selected to perform the initial diagnosis of the patient's biopsy specimens using a blinded method. For cases where there were diagnostic disagreement, the final diagnosis was made by the pathologist from Jilin University. TNM staging was determined by the treating physician responsible for the patient. Moreover, all visit records were selected from the hospital's electronic medical record system. Patients who lacked treatment records and were treated at an outside institution, were still considered to be eligible for inclusion, assuming they could provide a specific treatment plan. All patients were recommended and received tumor-specific chemotherapy, including endocrine or targeted regimens. What’s more, to be consistent with SEER records, only patients who underwent radical tumor surgery were considered surgical cases. Partial excision is also a surgical case. Exclusion criteria were as follows: (1) patients with incomplete or unavailable clinicopathological and survival data (Absence, NA or unknow); (2) patients with one follow-up period of fewer than 3 years; and (3) patients with other primary tumor diseases.All methods were performed in accordance with the relevant guidelines and regulations (Declaration of Helsinki).

### Variable processing

Based on the previously pulled data, the cut-off values of the continuous variables (age, tumor size) were calculated using the X-tile, and these variables were transformed into categorical variables. The primary site was classified as Axis bone and Limb bones. The remaining low-incidence sites were classified as other. The number of primary tumors (Sequence number) was defined as only one and more. The tumor staging criteria was based on the 2010 edition of the American Joint Committee on Cancer (AJCC) guidelines [11].

### Development and validation of predictive models

The differences in survival time and clinical parameters between the training and validation groups were analyzed using independent samples t-test and chi-square test, respectively. Heat map was employed to show the Pearson’s correlation between the variables. Kaplan-Meier survival curve for OS estimation.

Univariate and multivariate cox proportional risk models were used to calculate hazard ratios (HRs) and 95% confidence intervals (CIs) for the training set and to determine the effect of predictors on OS. Independent risk factors identified by multivariate cox analysis offered the possibility to achieve the Nomogram predicting 1-year and 3-year survival in ES patients. Furthermore, a web calculator had been created to meet the possible digital needs of users. The calibration curves were used to assess the similarity of the actual outcomes to the predicted results of the Nomogram. Receiver operating characteristic (ROC) curves and areas under the curves (AUCs) at 1-year and 3-year were generated to evaluate prognostic accuracy. The training set was subjected to multiple validations, including internal validation of the SEER database and external validation of the multicenter data. Finally, a clinical decision curve evaluated the clinical utility value of the model. Statistical analysis was performed using R (version 4.0.5, https://www.r-project.org/).*P* values <0.05 was considered statistically significant.

## Result

### Transformation of variables

**Fig. 1:**
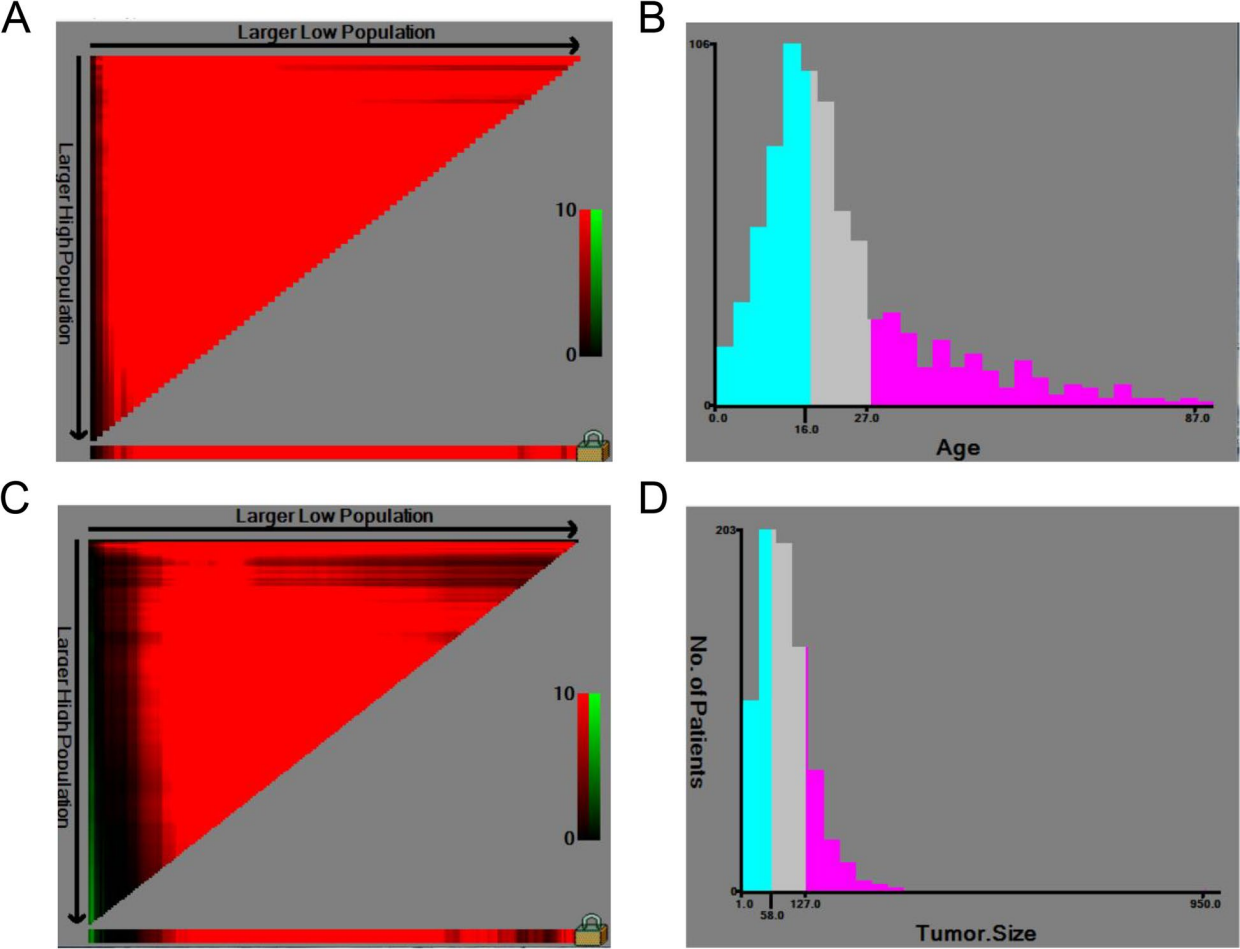
The upper line shows the cut-off value for age (**A**, **B**) and the lower line shows the cut-off value for tumor size (**C**, **D**)

Figure 1 showed the results of classifying two sets of continuous variables, age and tumor size, into three groups using X-tile software. In the right triangle on the left side of the A and C plots, the color of each pixel indicated the strength of the association of each cut point with survival, ranging from low (dark, black) to high (green, red), with red being negatively associated with survival and green representing a direct association. The horizontal x-axis represented all potential cut points from low to high (left to right) defined as the low subset, the vertical y-axis represented all potential cut points from high to low (top to bottom) for the high subset, and the slanted edge defined all potential cut points for the middle subset, with arrows indicating the direction of increasing size for the low (x-axis) and high subsets (y-axis). The best cut point appeared in the brightest pixel (red) identified by the computer software. At the same time, it indicated a negative correlation with the survival rate.

The x-tile was used to select the best cut point by choosing the highest x2 value, with specific values of 16 and 27 in Figure B and 58 and 127 in Figure C.

### Patient demographics

In total, 818 ES patients were included in the study, 767 from the SEER dataset. Of the 65 patients in validation sets from multiple centers, 14 were filtered out, with 4 cases refusing chemotherapy, 2 cases with malignant tumor progression dying at the time of hospitalization, and 8 with less than 3 years of follow-up. The remaining 51 patients were enrolled in the study. The patients’ demographics were presented in Table 1. The majority of patients were adolescents under 17 years (379 cases, 46.33%), and most patients underwent chemotherapy (776, 94.87%). Of these cases, 111 patients (13.57%) with bone metastatic were identified (Table 1).

**Table 1:** Baseline data of Ewing's sarcoma patients

Variable	level	SEER data(Training group, *N*=767)	Multicenter data(validation group, *N*=51)	p
Survival time (mean (SD))	NA	30.98 (22.73)	29.71 (22.40)	0.698
Age (%)	<17	354 (46.2)	25 (49.0)	0.41
	17-27	229 (29.9)	11 (21.6)	
	>27	184 (24.0)	15 (29.4)	
sex (%)	Male	439 (57.2)	28 (54.9)	0.857
	Female	328 (42.8)	23 (45.1)	
Primary.Site (%)	Axis bone	256 (33.4)	13 (25.5)	0.281
	Limb bone	320 (41.7)	27 (52.9)	
	Other	191 (24.9)	11 (21.6)	
Laterality (%)	Left	301 (39.2)	21 (41.2)	0.832
	Right	257 (33.5)	15 (29.4)	
	Not a paired site	209 (27.2)	15 (29.4)	
T (%)	T1	328 (42.8)	20 (39.2)	0.081
	T2	398 (51.9)	25 (49.0)	
	T3	23 (3.0)	5 (9.8)	
	TX	18 (2.3)	1 (2.0)	
M (%)	M0	536 (69.9)	30 (58.8)	0.134
	M1	231 (30.1)	21 (41.2)	
Radiation (%)	No	382 (49.8)	29 (56.9)	0.406
	Yes	385 (50.2)	22 (43.1)	
Chemotherapy (%)	No	42 (5.5)	0 (0.0)	0.165
	Yes	725 (94.5)	51 (100.0)	
Tumor size (%)	≤58	242 (31.6)	15 (29.4)	0.503
	59-128	378 (49.3)	29 (56.9)	
	>127	147 (19.2)	7 (13.7)	
Bone metastases (%)	No	667 (87.0)	40 (78.4)	0.131
	Yes	100 (13.0)	11 (21.6)	
Lung metastases (%)	No	628 (81.9)	41 (80.4)	0.937
	Yes	139 (18.1)	10 (19.6)	
surgery (%)	No	305 (39.8)	25 (49.0)	0.247
	Yes	462 (60.2)	26 (51.0)	

**Fig. 2:**
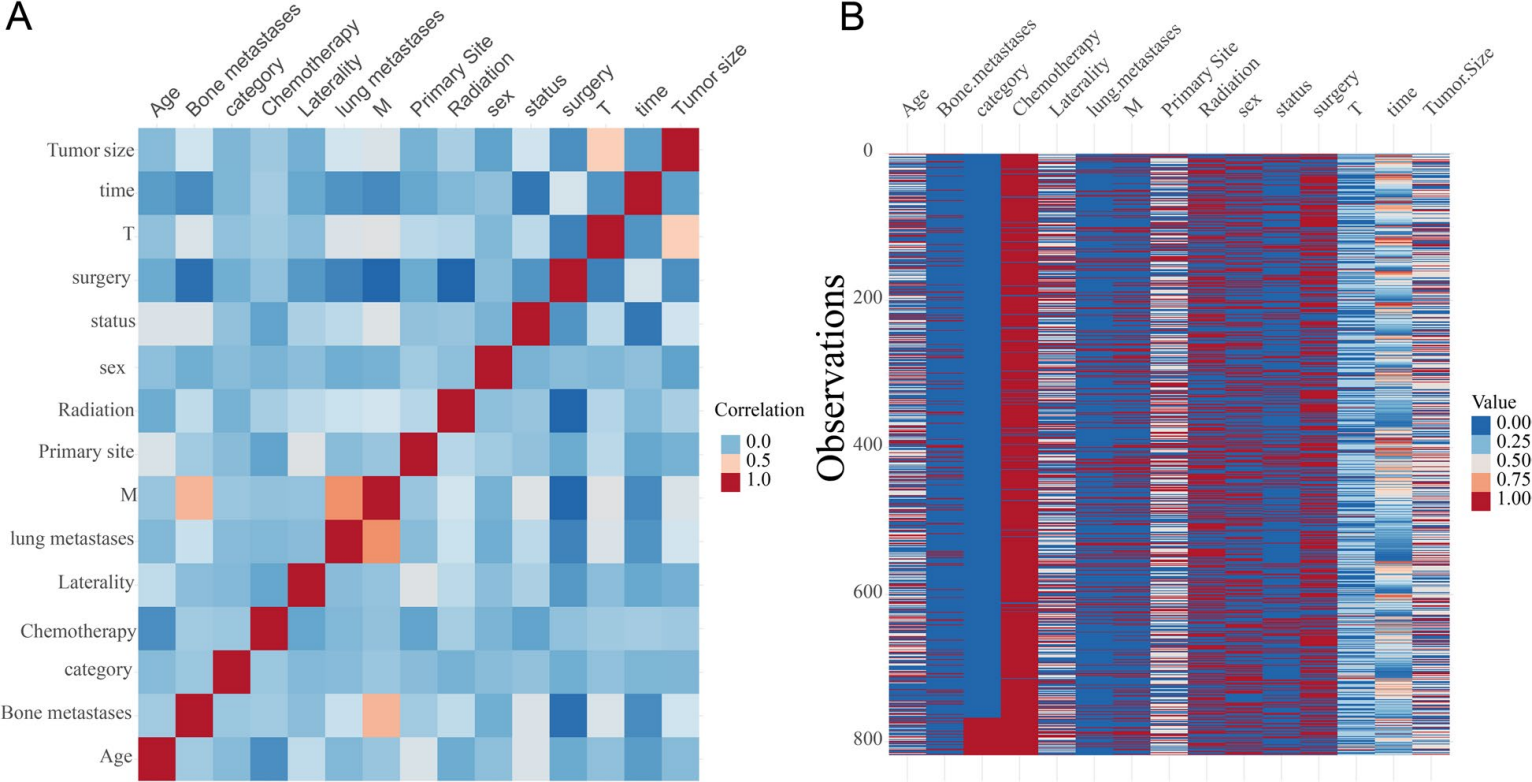
Heat map about the correlations of the included variables

In the correlation plot (Fig. 2), the factor groups with significant negative correlations were surgery and bone metastasis, surgery and M stage, surgery and radiotherapy, and time and status. The factor groups with significant positive correlations were T stage and tumor size, M stage and bone metastasis, M stage and lung metastasis.Besides, chemotherapy and time and status correlated remarkably.

### The risk factors of prognosis in Ewing's sarcoma patients

Univariate and multivariate cox regression analyses were performed to identify potential risk factors for the prognosis of ES. Five independent risk factors and two independent protective factors were shown to be associated with OS in ES, including age, chemotherapy, tumor size, bone metastases (Table 2). Survival risk was higher in older age, tumors larger than 58 mm, presence of bone metastases, laterality.

**Table 2:** Univariate and multivariate cox regression analysis of Ewing's sarcoma

	Univariate Cox		Multivariate Cox	
Characteristics	HR (CI 95%)	P	HR (CI 95%)	P
**Age**				
	Ref	Ref	Ref	Ref
17-27	2.048 (1.419-2.956)	<0.001	1.856 (1.279 - 2.695)	<0.01
	3.615 (2.531-5.163)	<0.001	4.036 (2.750 – 5.924)	<0.001
**Sex**				
Female	Ref	Ref	Ref	Ref
Male	0.907 (0.680-1.211)	0.509	NA	NA
**Primary Site**				
Aix bone	Ref	Ref	Ref	Ref
Limb bone	1.459 (1.042-2.044)	<0.05	1.091 (0.742 - 1.605)	0.658
other	1.332 (0.904-1.962)	0.147	0.821 (0.522 - 1.290)	0.392
**Laterality**				
Left	Ref	Ref	Ref	Ref
Not a paired site	1.659 (1.175-2.344)	<0.01	1.447 (0.981 – 2.134)	0.062
right	1.255 (0.883-1.782)	0.205	1.166 (0.811 - 1.675)	0.408
**T**				
T1	Ref	Ref	Ref	Ref
T2	1.748 (1.279-2.387)	<0.001	0.766 (0.501 - 1.173)	0.221
T3	3.048 (1.457-6.378)	<0.01	0.867 (0.387 - 1.939)	0.727
TX	4.109 (1.873-9.015)	<0.001	1.307 (0.552 - 3.095)	0.543
**M**				
M0	Ref	Ref	Ref	Ref
M1	3.37 (2.536-4.484)	<0.001	1.538 (0.940 - 2.518)	0.087
**Radiation**				
No	Ref	Ref	Ref	Ref
Yes	1.284(0.965-1.710)	0.087	NA	NA
**Chemotherapy**				
No	Ref	Ref	Ref	Ref
Yes	0.411 (0.253-0.669)	<0.001	0.374 (0.222 - 0.630)	<0.001
**Tumor Size**				
≤58	Ref	Ref	Ref	Ref
59-128	2.301(1.548-3.420)	<0.001	2.656(1.643-4.292)	<0.001
	3.532(2.283-5.465)	<0.001	3.900(2.174-6.996)	<0.001
**Bone metastases**				
No	Ref	Ref	Ref	Ref
Yes	3.808 (2.753-5.267)	<0.001	2.228 (1.419- 3.500)	<0.01
**Lung metastases**				63
No	Ref	Ref	Ref	Ref
Yes	2.502 (1.824-3.431)	<0.001	1.386 (0.879 - 2.186)	0.160
**Surgery**				
No	Ref	Ref	Ref	Ref
Yes	0.406(0.305-0.541)	<0.001	0.731 (0.526 – 1.016)	0.62

**Fig. 3:**
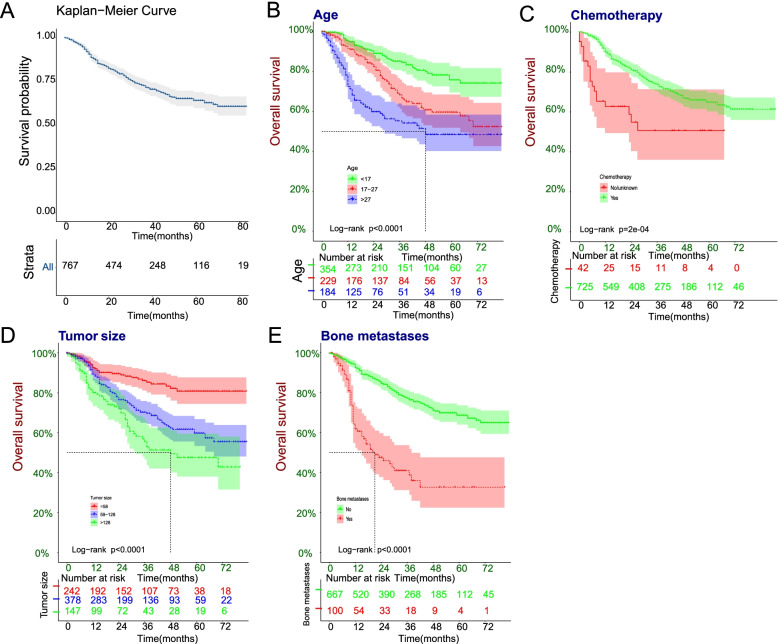
Kaplan-Meier survival curve in the training set

Kaplan Meier survival curves were further plotted for the ES patients, and independent risk factors were obtained from multivariate cox analysis. Log-rank test was performed in the training set, and the results showed that the *p*-values of the four independent risk factors (age, bone metastases, chemotherapy, and tumor size) were less than 0.05 (Fig. 3). The same results of bone metastases, was also statistical significance in the validation set (*p* <0.05) (Figure S1).

### Establishment of Nomogram and web calculator

**Fig. 4:**
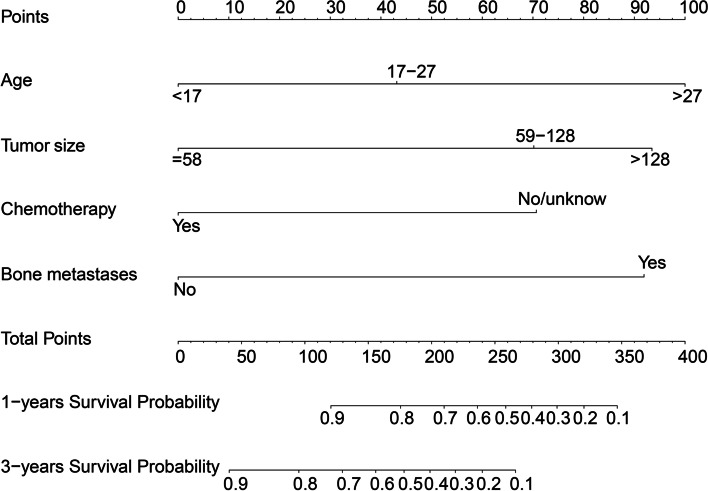
Nomogram for predicting 1 - and 3-year survival for patients with Ewing sarcoma

Based on the results of cox analysis, a nomogram was constructed to predict 1-year and 3-year OS of ES patients (Fig. 4), containing predictors such as age, tumor size, bone metastasis, and chemotherapy. Furthermore, an online web calculator was designed (https://drwenleli0910.shinyapps.io/EwingApp/).

### Calibration degree of the model

**Fig. 5:**
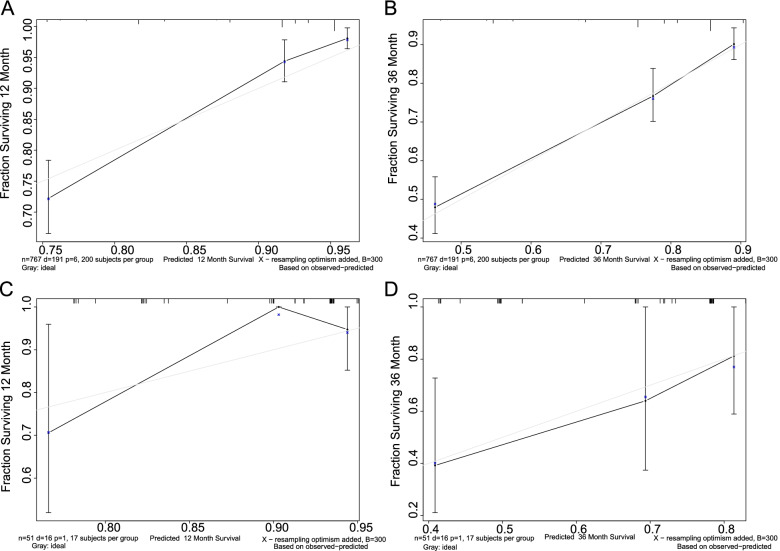
**A** and **B** are calibration plots of 1-year and 3-year survival rates for the training set; **C** and **D** are calibration plots of 1-year and 3-year survival rates for the validation set

The calibration plots were based on actual and predicted incidence. To facilitate comparison, a grey line had been added to the graph, representing y=x, which meant that the predicted and actual incidences were the same exactly. Therefore, the closer the grey line and the black line indicated the closer the predicted and actual incidence rates were, the better the model recognition ability was. In Fig 5, both internal validation (A and B) and external validation (C and D) showed a good recognition ability.

### Predictive power and discrimination of the model

**Fig. 6:**
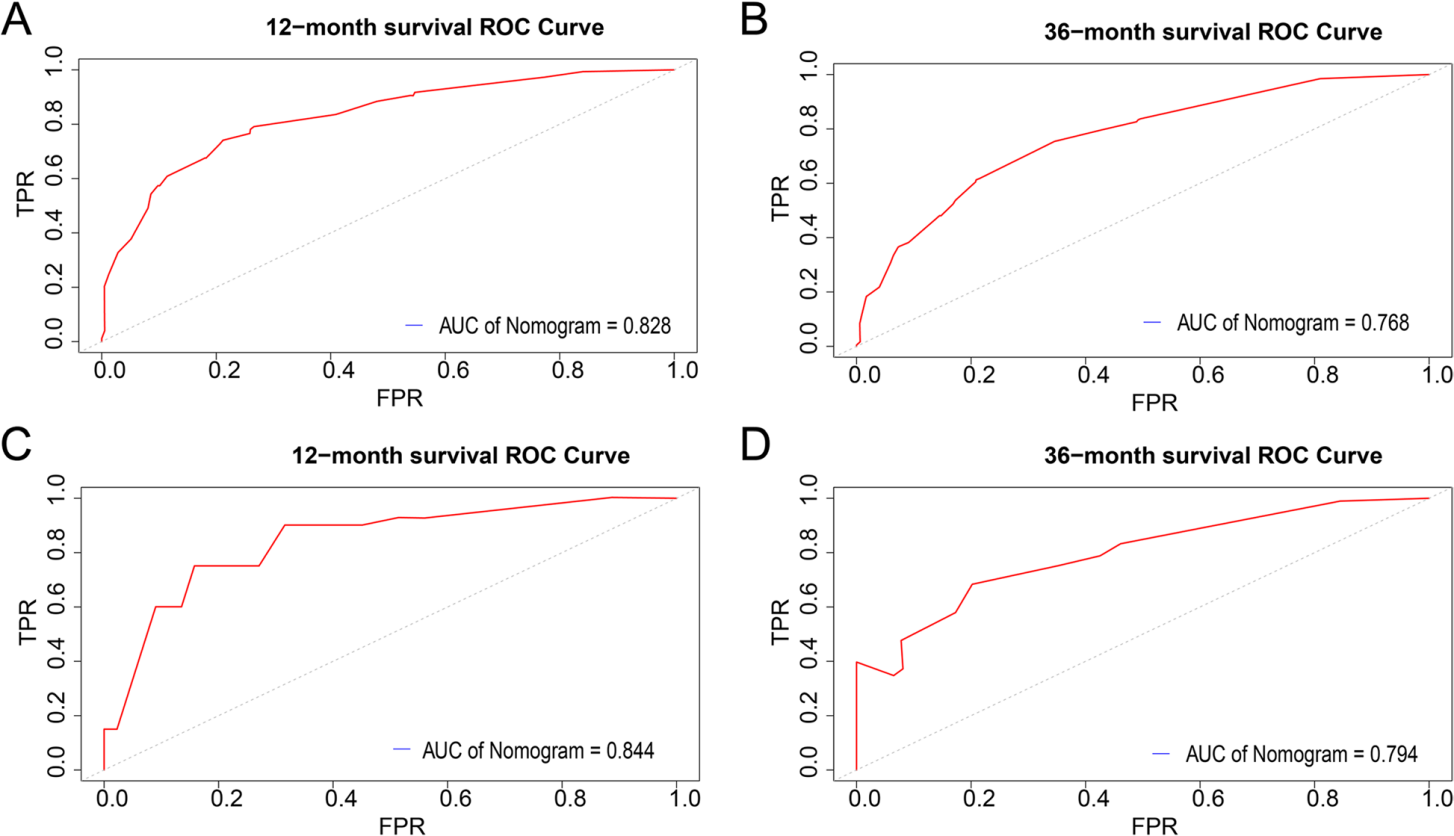
**A**, **B** showed the 1-year and 3-year ROC curves for the training group. **C**, **D** showed the 1-year and 3-year ROC curves for the validation group

An area under the ROC curve (AUC) greater than 0.5 proved that the model had some predictive ability. Meanwhile, the closer the area under the ROC curve was to 1, the better the prediction ability of the model. Figure 6A, B showed the internally validated ROC curves for 1-year and 3-year survival rates, with AUC values of 0.828 and 0.768, respectively. Figure 6C, D showed the externally validated ROC curves for 1-year and 3-year survival rates, with AUC values of 0.844 and 0.794, respectively. It showed that the model had a excellent predictive ability in predicting patients' 1-year survival rates and possessed better predictive ability in 3-year survival rates.

### Clinical usefulness of the model

The answer to whether model-assisted decision-making improved patients’ prognosis lies in prospective assessment randomly assigning patients to model-based or non-model-based decisions and comparing outcomes. However, this is not realistic, so decision curves analysis (DCA) have been introduced to assess whether model-assisted decision-making improved patient outcomes.

**Fig. 7:**
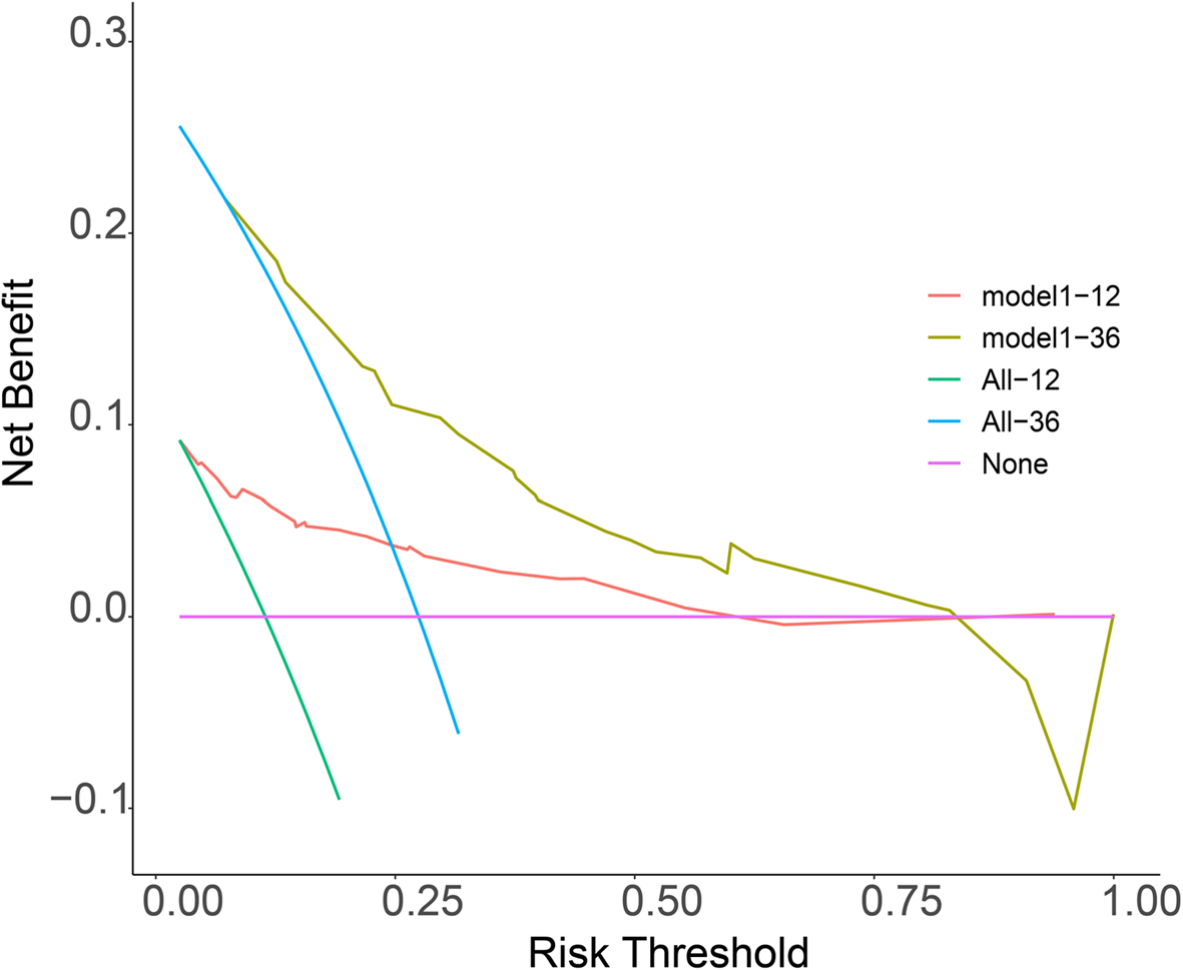
Decision curves analysis (DCA) for 1-year survival(Red line) and 3-year survival( brown line)

Figure 7 plotted the DCA for patients’ survival at 1 and 3 years, where x = risk threshold, i.e., the probability of triggering a medical intervention, and y = the net benefit received by implementing the treatment at this probability. The purple horizontal line (None) represented all patients who did not trigger the intervention, the Green and blue line (All) all or both triggered the intervention, and the red and brown line were the column line graph guiding the triggering of one medical intervention, which could be seen as a net benefit of 0 for purple, and at the same threshold probability (x). It was clear that brown line received a higher net benefit than blue and red line received a higher net benefit than green. In general, the further the model curve was from the x, y axis, the stronger its clinical utility. Figure 7 showed that the model had a good clinical utility in improving both 1-year and 3-year survival in ES patients, with a slightly better net gain at 3 years than at 1 year.

## Discussion

A previous studies had shown that advanced age, larger tumor size, distant metastasis and specific tumor primary sites (axial bone) might deteriorate patient prognosis, and tumor metastasis was defined as the most important factor affecting patient survival [12]. However, no internationally recognized risk classification scheme for ES patients had been formed. For non-metastatic ES patients, the use of multiple treatment modalities such as surgery, radiotherapy and chemotherapy had increased long-term survival rates from 10%-15% to 60%-70% [13, 14]. Temozolomide/irinotecan regimen proved to be the most effective, with 47% of ES patients in remission [15]. On the other end, approximately 80% of patients with metastases had a discouraging survival outcome [16, 17]. The most predominant site of distant metastases was lung (70-80%), followed by bone (40-45%) [18]. Recently, it had been reported that neoadjuvant chemotherapy in combination with surgery and radiation therapy had the potential to achieve local control of metastatic tumors and improve patient prognosis [19]. Therefore, reliable early assessment of metastatic risk was critical to determine which patients were truly at higher risk so that they could be taken into consideration for neoadjuvant therapy. The Nomogram had shown its unique advantages in the field of prediction. The probability of event occurrence generated by this prediction model reflected the fact that researchers consider multiple predictors related to the individual rather than assessing statistically positive variables in isolation. At the same time, the breadth of application and ease of use of the Nomogram had provided considerable assistance to healthcare and had been widely used as a result, including in ES [9, 20].

In the current study, four independent prognostic factors were identified and integrated into the established Nomogram, including age, tumor size, bone metastasis, and chemotherapy,

Among 3 manually divided age intervals, adolescents (<17 years, 46.27%) represented the most common category of patients with Ewing sarcoma. The peak incidence of Ewing's sarcoma reported by Grünewald et al. was 15 years which was in agreement with our findings [21]. However, the prognosis of tumors were not completely synchronized with the incidence, and older patients were more at risk of poor prognosis [17, 22]. This was due to the older patients having more comorbidities, including diabetes, hypertension and other cancers, and were less tolerant of the treatment modalities, and clinicians tended to choose more conservative initial treatment strategies, and patients therefore took higher risks [23]. Worch and colleagues showed that the lower metastasis rate might be responsible for the better survival of younger patients, and found significant differences in the sites of primary and metastatic tumors with age [24] .

We found that patients with N1 with regional lymph node involvement or NX with unclear regional lymph node status had a lower overall survival rate compared to patients without regional lymph node involvement. Furthermore, Applebaum et al suggested that patients with extraosseous primary tumors had a significantly higher rate of regional lymph node metastasis [25]. Therefore, we considered it necessary to emphasize the examination of regional lymph nodes in diagnosed or suspected Ewing sarcoma, including radiological and pathological biopsies.

Compared to our finding of a tumor size threshold of 5.8cm, one study reported that Ewing sarcoma larger than 8cm had a poorer prognosis and a significantly greater likelihood of recurrent tumor enlargement independent of measurement and treatment [17, 26]. Larger tumor size and axial site of origin might be associated with metastatic disease, both of which had turned out to be risk factors for reduced survival [27, 28]. In contrast, Li et al did not conclude that tumor size affected ES survival. This might be linked to that the tumor had already shrunk due to effective chemotherapy/radiotherapy at the time of measurement [29].

ES was extremely sensitive to radiation compared to other primary osteosarcomas. The benefit of radiotherapy in local control had been shown, especially for tumors larger than 200 ML in volume and post-chemotherapy necrosis [21]. Meanwhile,ES undergoing surgical resection, whether primary or metastatic, had a highly statistically significant prolonged survival [30]. Combination chemotherapy regimens using vincristine, adriamycin, cyclophosphamide and actinomycin increase survival in ES patients from approximately 10% to 70-80% [31]. In fact, the subgroup of patients who underwent extensive surgical resection and induction chemotherapy achieved the greatest survival benefit [21]. However, the SEER database did not contain detailed chemotherapy regimens and only suggested whether patients had received chemotherapy. Prediction of survival for specific drugs needed to be further investigated.

Lung and bone were the preferred sites for metastatic Ewing sarcoma, but the two represented different prognoses for patients. In general, Ewing sarcoma with lung metastases alone get a better prognosis than metastases from bone alone or multiple metastases from more than two sites. Our finding of statistically significant patients with bone metastases also endorsed this view. Differential overall survival was shown to be probably closely related to the interaction of tumor cells with the microenvironment of bone, which basic fibroblast growth factor secreted by bone marrow stromal cells promotes ES cell metastasis [32].

The origin of the sarcoma was subdivided into primary site and laterality in this study. The primary site refers to the axial bone, extremity bone, and others. Laterality refers to left, right and not a paired site. Statistical significance was found only in laterality, with the possible explanation that laterality includes patients with pelvic Ewing sarcoma who not covered in the primary site classification. ES of the pelvis has an insidious onset, frequent local spread, and complex anatomy. In a considerable number of patients, the tumor size that was already considerable at the time of initial diagnosis indicated an advanced stage [29]. Radical tumor surgery and regional radiation therapy were difficult to achieve, so patients with ES of the pelvis had an unpromising chance of survival [33]. Subsequent studies were called upon to reveal more about the impact of laterality on the prognosis of ES.

This study envisioned a scenario in which surgeons applied predictive models in their work: when communicating with ES patients about their condition and explaining treatment modalities, patients with no specialized background could likewise understood the necessity and feasibility of treatment to a greater extent with the advantage of Nomogram visualization. The ultimate goal of clinical predictive modeling was to help surgeons make medical decisions that improved patient prognosis and reduced medical costs.

There are several limitations to our study. First, the data from the SEER database and independent validation both were retrospective, which could result in potential selection bias. Second, a few candidate prognostic variables, surgical margin status, vascular invasion, surgical treatment details, and specific radiotherapy and chemotherapy modalities were unavailable. These variables should be further investigated to improve the accuracy of the prediction models. In addition, the presence of missing follow-ups had diluted our sample size, which might have an adverse effect on the conclusions. Future studies should concentrate on using a prospective cohort study design to improve the credibility of the findings. Machine learning algorithms played an indispensable role in the exploration of artificial intelligence in medicine, especially in oncology-related frontiers [34]. Further research could not only focus on using optimized designs such as prospective cohort studies but also attempt to adopt machine learning to investigate Ewing sarcoma.

## Conclusion

The prognosis for patients with ES was unacceptable, and the predominantly adolescent patient population required a multidisciplinary team to develop individualized diagnostic and treatment protocols, including radiologists, pathologists, orthopedic surgeons, pediatricians, and oncologists. This study combined data from the SEER database and four independent medical centers to assess the predictors affecting survival in ES. The proposed Nomogram contributes to guide treatment, follow-up, and improving treatment accuracy and individualization.

## Supplementary Information

**Additional file 1: Figure S1.** Kaplan-Meier survival curve in the validation set.

## Data Availability

The dataset from SEER database generated and/or analyzed during the current study are available in the SEER dataset repository (https://seer.cancer.gov/).
