# Supplementary material for: A tool for predicting overall survival in patients with Ewing sarcoma: a multicenter retrospective study
Source: BMC Cancer. 2022 Aug 23;22:914. doi: 10.1186/s12885-022-09796-7 (PMC9400324; doi:10.1186/s12885-022-09796-7)
Supplement: Supplementary file 1 — Additional file 1: Figure S1. Kaplan-Meier survival curve in the validation set. [file 12885_2022_9796_MOESM1_ESM.zip › Supplementary materials.doc]

**Supplementary materials**

**Figure S1.** Kaplan-Meier survival curve in the validation set.
